# Finite element fracture load analysis and dark-field X-ray imaging of osteoporotic and healthy vertebrae in human lumbar spine specimens

**DOI:** 10.1186/s12891-025-08709-6

**Published:** 2025-06-03

**Authors:** N. Hesse, D. Strack, J. F. Rischewski, F. T. Gassert, A. Kufner, T. Urban, M. E. Lochschmidt, B. J. Schwaiger, C. Braun, D. P. Mueller, D. Pfeiffer, T. Baum, K. Subburaj, F. Pfeiffer, A. S. Gersing

**Affiliations:** 1 Department of Radiology, University Hospital, LMU Munich, Ziemssenstr. 5, Munich, 80336 Germany; 2 Department of Mechanical and Production Engineering - Design and Manufacturing, Aarhus University, Katrinebjergvej 89 G-F, Aarhus N, 8200 Denmark; 3 Department of Diagnostic and Interventional Radiology, Klinikum Rechts Der Isar, School of Medicine and Health, Technical University of Munich, Ismaninger Straße 22, Munich, 81675 Germany; 4 Institute of Legal Medicine, Faculty of Medicine, LMU Munich, Nußbaumstraße 26, Munich, 80336 Germany; 5 Department of Diagnostic’ and Interventional Neuroradiology, School of Medicine and Health, Klinikum Rechts Der Isar, Technical University of Munich, Ismaninger Straße 22, Munich, 81675 Germany; 6 Munich Institute for Advanced Study, Technical University of Munich, Lichtenbergstraße 2a, Garching, 85748 Germany; 7 Institute for Diagnostic and Interventional Neuroradiology, University Hospital, LMU Munich, Marchioninistraße 15, Munich, 81377 Germany; 8 Department of Trauma Surgery, Klinikum Rechts Der Isar, School of Medicine and Health, Technical University of Munich, Ismaninger Straße 22, Munich, 81675 Germany; 9 Department of Physics, Chair of Biomedical Physics, School of Natural Sciences, Technical University of Munich, James-Franck-Str. 1, Garching, 85748 Germany; 10 Munich Institute of Biomedical Engineering, Technical University of Munich, Boltzmannstraße 11, Garching, 85748 Germany; 11 Department of Radiology, University of California San Francisco, 505 Parnassus Street, San Francisco, CA 94143 USA

**Keywords:** Finite element analysis, Dark-field imaging, Osteoporosis, Spine, Bone

## Abstract

**Purpose:**

This study investigated the association of measurements from a clinical X-ray dark-field prototype system and CT-based finite element analysis (FEA) in lumbar spine specimens.

**Materials and Methods:**

In this prospective study, human cadaveric spine specimens (L2 to L4) were examined using a clinical prototype for dark-field radiography, yielding both attenuation and dark-field images. Specimens were scanned in vertical and horizontal positions. Volumetric bone mineral density (BMD) values were derived from quantitative CT measurements. Bone segmentation masks derived from CT-images were used for FEA-estimated fracture load (FL) calculations. FEA-estimated FL, dark-field, and attenuation signals were compared between osteoporotic/osteopenic (BMD < 120 mg/cm^3^) and non-osteoporotic/osteopenic specimens using the paired t-test and the Wilcoxon Mann–Whitney U test. Associations were tested using Spearman correlation.

**Results:**

Fifty-nine vertebrae from 20 lumbar spine specimens (mean age, 73 years ± 13; 11 women) were studied. FEA-estimated FL correlated with BMD (r = 0.75, *p* < .001) and was significantly lower in osteoporotic/osteopenic vertebrae (1222 ± 566 vs. 2880 ± 1182, *p* < .001). Dark-field and attenuation signals were positively correlated with FEA-estimated FL, in both vertical (r_darkfield_ = 0.64, *p* < .001, r_attenuation_ = 0.82, *p * < .001) and horizontal position (r_darkfield_ = 0.55, *p* < .001, r_attenuation_ = 0.81, *p* < .001).

**Conclusion:**

Dark-field and attenuation signals assessed using a clinical X-ray dark-field system significantly correlated with FEA-estimated FL in human spine specimens with and without osteoporosis/osteopenia. Dark-Field imaging may complement existing assessment methods for bone strength as a dose-efficient, accessible tool.

## Introduction

Osteoporosis is characterized by reduced bone mass and strength, which increases the risk of fragility fractures [1]. Furthermore, osteoporosis is a major health problem in our aging society due to the socioeconomic burden on the healthcare system and the contribution of fragility fractures to morbidity and disability-adjusted years of life [2, 3]. The spine is one of the most common sites for fragility fractures and affected patients have more than a tenfold increased risk of additional future vertebral fractures [4–6]. Thus, the assessment of bone strength is crucial, as the treatment and prevention of osteoporosis reduce the risk of fractures and subsequent complications [7].

Clinical routine assessment of bone structure relies on attenuation-based radiographic techniques — dual-energy X-ray absorptiometry (DXA) and quantitative computed tomography (qCT) — to measure bone mineral density (BMD) [8, 9]. While DXA, measuring areal BMD, is still considered the clinical standard of reference, it has been found to be inadequate for identifying patients at high risk of fractures [10, 11]. qCT, measuring volumetric BMD, has emerged as a comparable alternative to DXA, with previous studies indicating a higher sensitivity for detecting osteoporosis [12]. However, qCT has notable drawbacks, including a lack of standardization, increased radiation exposure, and higher costs [13]. Furthermore, BMD derived from qCT neither fully explains fracture incidence [14, 15].

Bone strength is determined by both BMD and bone quality, with BMD contributing approximately 70% to overall bone strength [16]. Grating-based X-ray dark-field imaging has been introduced as a new and promising technique for the assessment of bone quality ex-vivo [17–22]. Dark-field imaging relies on a Talbot-Lau interferometer consisting of three gratings (G0, G1, and G2) positioned between a conventional X-ray source and detector. Unlike conventional radiography, which measures only absorption (attenuation), dark-field imaging additionally captures the ultra-small-angle scattering occurring at the material interfaces within the specimen under investigation on the submicrometer- or micrometer-length scale below the actual pixel size [23]. In previous ex-vivo studies dark-field imaging has been shown to provide structural information about the trabecular alignment and the microstructure of vertebral bone at low radiation dose [18–21]. Furthermore, the ability of dark-filed imaging to differentiate between healthy and osteoporotic vertebrae was demonstrated [20]. Current dark-field prototypes operate at dose levels comparable to standard chest X-ray examinations, typically lower than volumetric CT scans [21].

CT-based finite element analysis (FEA) is a computational technique that can simulate the mechanical behavior of bone using patient-specific 3D anatomical models. Initially developed and validated through ex-vivo experiments, FEA models are now increasingly applied in-vivo to estimated bone strength and fracture risk [24, 25]. In a FEA simulation, a compression loading condition is created by applying a displacement load to the upper surface. By analyzing the load versus displacement curve, fracture load (FL) and fracture displacement can be estimated [26]. The supremacy of CT-based FEA in predicting bone strength and vertebral fractures over the use of the reference standard BMD has been established in several studies [26–28].

The aim of the study was to investigate the association of dark-field radiography parameters and CT based FEA-estimated FL in human lumbar vertebrae with and without osteoporosis/osteopenia, exploring the potential of dark-field imaging for assessing bone strength.

## Methods

### Specimens

Institutional review board approval was obtained prior to this study (Ethics Commission: Medical Faculty of Technical University Munich, reference number 392/20 S). The study was conducted in accordance with the Declaration of Helsinki. As described previously, lumbar spine specimens (vertebra L2 to L4) from human cadavers with clinically indicated post-mortem autopsy were harvested within 24 h after death. Patients with previous spine surgery and known osseous metastases were excluded. From a single specimen, only L2 and L3 were harvested, resulting in a total of 59 vertebrae from 20 human donors [20].

### CT imaging and BMD measurements

Ex-vivo CT scans were performed using a dual-layer spectral CT system (IQon Spectral CT, Philips Healthcare) with the following parameters: collimation of 0.6 mm, pixel spacing of 0.3 mm, a spiral pitch factor of 0.39, a peak tube voltage of 120 kV, and a tube current of 347 mA. BMD values were derived from qCT examinations that were calibrated asynchronously [29, 30]. Hounsfield unit (HU) values for all lumbar vertebrae were obtained from representative median slices using the IDS7 PACS (Sectra AB, Linköping, Sweden). A radiologist (FTG, with four years of experience in musculoskeletal imaging) manually segmented regions of interest (ROIs) in the anterior section of the vertebrae in the sagittal plane [31]. Vertebrae with BMD values < 120 mg/cm^3^ were classified as osteoporotic/osteopenic, and BMD values ≥ 120 mg/cm^3^ as non-osteoporotic/osteopenic [32]*.*

### X-ray dark-field imaging and quantitative image evaluation

We used the prototype for clinical dark-field chest radiography consisting of conventional medical X-ray devices (tube, MRC 200 0508 ROT-GS 1003, Philips Medical Systems, Hamburg, Germany, detector, PIXIUM 4343 F^4^, Trixell, Moirans, France) in combination with a Talbot-Lau interferometer with three gratings [33, 34]. For the assessment of bone the setup’s sensitivity had to be increased by positioning the sample further away from the analyzer grating due to the low dark-field signal of bone [20, 21].

**Fig. 1:**
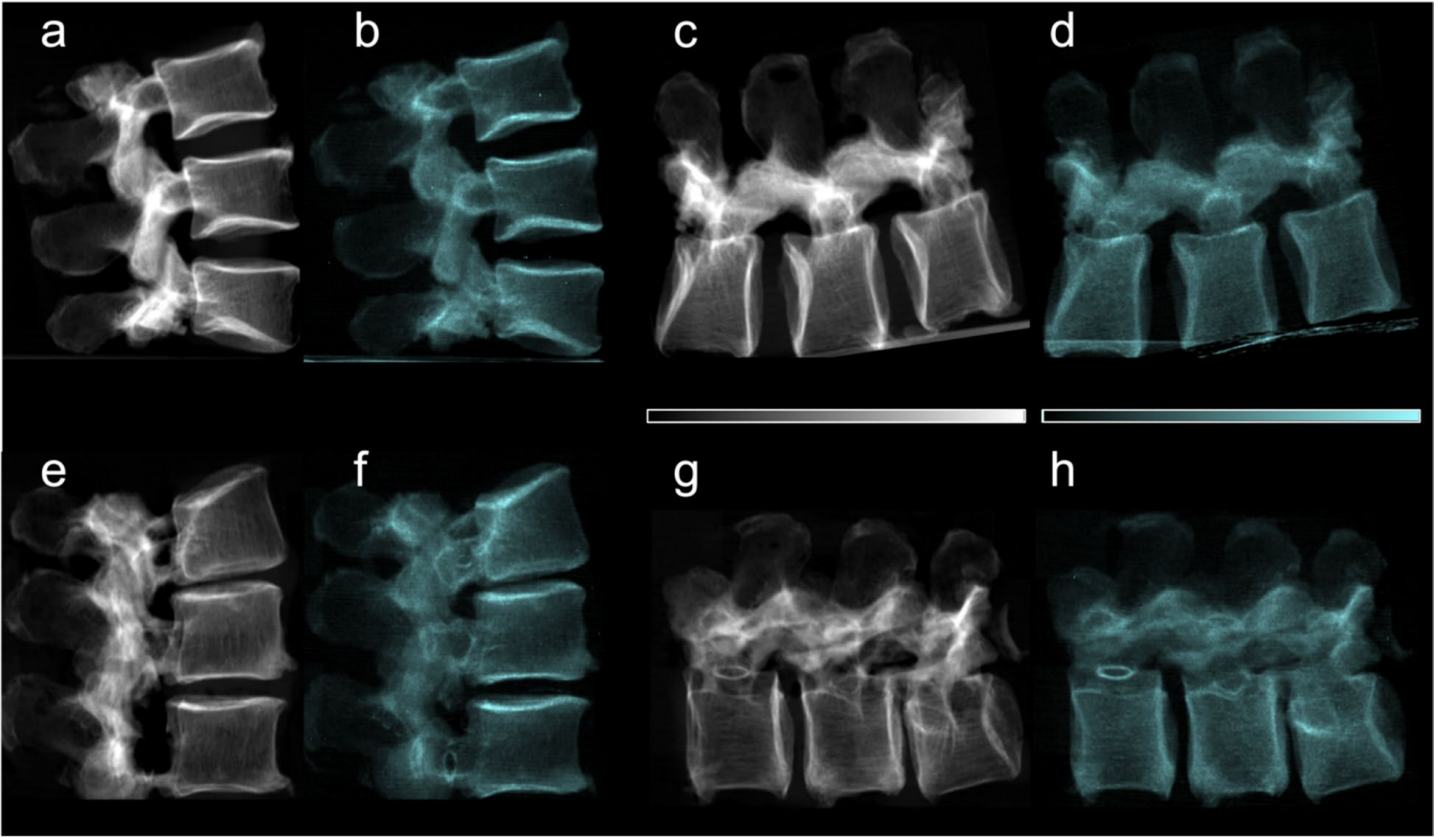
Lateral conventional (**a**, **c**, **e**, **g**) and co-registered dark-field (**b**, **d**, **f**, **h**) radiographs in vertical (**a**, **b**, **e**, **f**) and horizontal (**c**, **d**, **g**, **h**) position of the spine specimen of a 73-year-old man with normal BMD values (mean BMD = 201.9 mg/cm^3^) (**a**–**d**) and of a 78-year-old woman with osteoporotic BMD values (mean BMD = 46.3 mg/cm^3^) (**e**–**h**). Compared to the specimen without osteoporosis/osteopenia, the attenuation signal appears reduced in the osteoporotic specimen. While trabecular texture is visible in both attenuation images, it appears more sparse in the osteoporotic specimen. In the dark-field images, the trabecular bone signal is also lower in the osteoporotic specimen, though the difference is less pronounced. The trabecular structure seen in the attenuation images is not visible in the dark-field images, and if present, it appears very faint

The specimens were imaged in a lateral position within a water bath to minimize the impact of air surrounding the sample. To reduce the impact of Compton scatter and beam hardening, reference scans were conducted using a water container without the specimen. Additionally, aluminum was used as an equivalent absorber material to apply beam hardening correction [35]. The reported values for the attenuation and dark-field signals correspond to the intensity (attenuation) and visibility (dark-field) of the signal relative to the measured intensity in water, presented on a logarithmic scale. For example, a visibility/intensity value of 0 in the sample indicates a signal strength equivalent to that measured in water, while a visibility value of 1 corresponds to a signal strength of 1/e compared to water. Due to the setup’s limited sensitivity to horizontally aligned structural elements parallel to the grating lamella, specimens were scanned in two positions: vertically to assess trabecular structures in the lateral orientation and horizontally to assess trabecular structures in the craniocaudal orientation [18–20]. To assess the influence of trabecular orientation, we also computed the ratio of the horizontal-to-vertical signals for each vertebra. Figure 1 shows example attenuation radiographs and dark-field radiographs.

For quantitative image evaluation attenuation images in vertical and horizontal position were co-registered with a rigid registration using Phyton and SimpleElastix. ROIs were segmented manually in the anterior part of the vertebra on the overlay images, excluding sclerotic and superimposed areas. Registration accuracy was assessed using color-coded overlays. The same ROIs were then applied to both the attenuation and dark-field images. Quantitative values were calculated by averaging the signal within each ROI [20].

### Finite element analysis

**Fig. 2:**
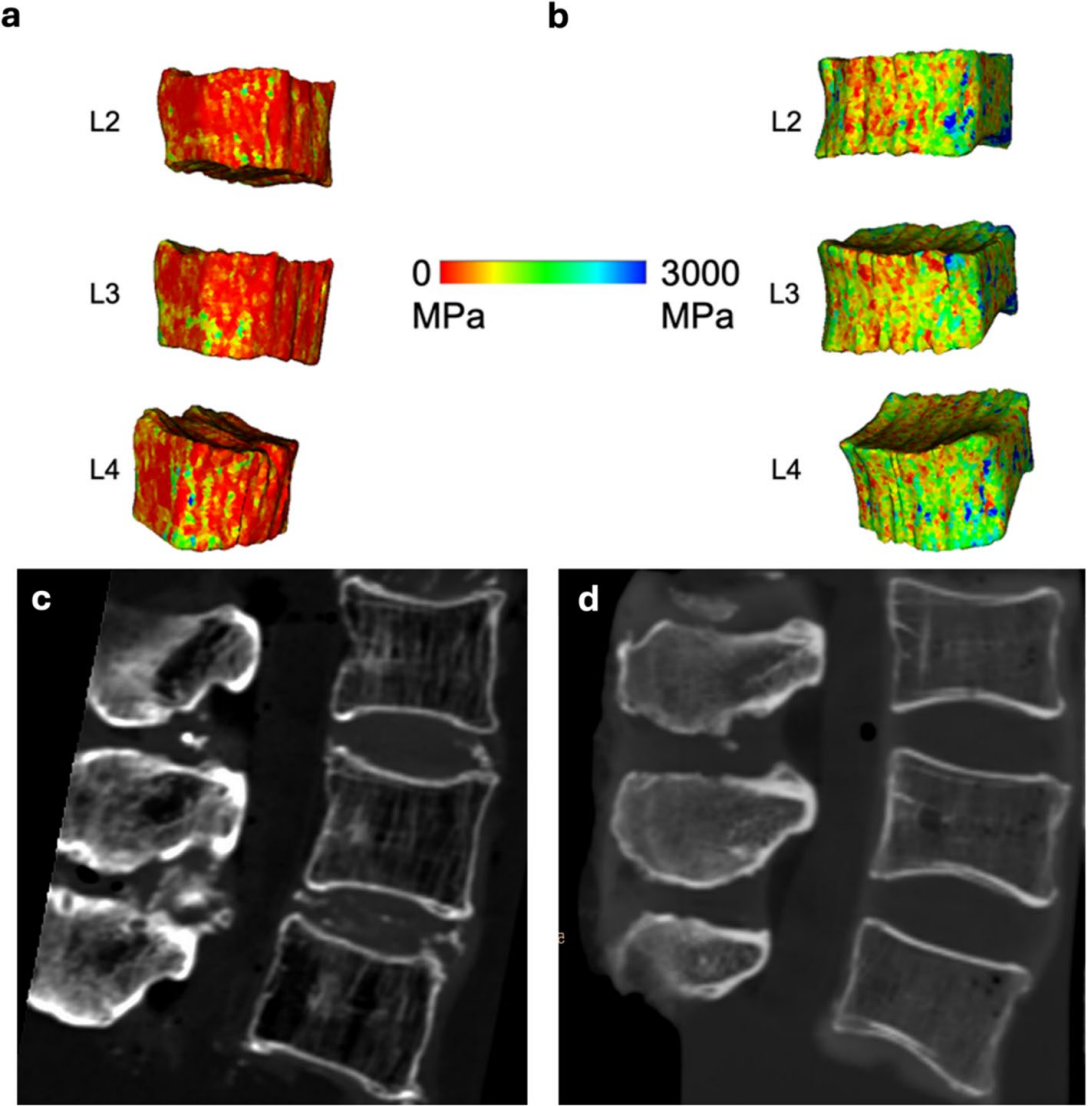
Patient-specific material mapped finite element meshes. Colors represent Young’s modulus, with a range adapted to highlight differences between subjects. **a** Osteoporotic vertebrae with visible lower mapped Young’s modulus (represented by red color, mean BMD 46.3 mg/cm^3^). **b** Vertebrae from a healthy subject with higher mapped Young’s modulus (represented by green color, mean BMD 201.9 mg/cm^3^). **c** and **d** show representative sagittal CT images of the specimens

FEA was carried out based on CT-derived geometries of the vertebrae to calculate the respective FL. Segmentations of the vertebrae were obtained semiautomatically using thresholding combined with gap-filling and manual post-processing. Based on the segmented geometry of the vertebrae, a three-dimensional model was reconstructed and meshed with linear tetrahedral elements. Based on a mesh convergence analysis, a mesh element length of 1.25 mm was chosen and utilized in the Abaqus CAE Environment (version 2021, Dassault Systems, Johnston, RI, USA). Engineers conducting the FEA were blinded regarding the osteoporosis status of the specimens. After meshing the vertebral geometry, subject-specific non-linear material parameters were assigned to the mesh using Bonemat V3.2 (http://www.bonemat.org/) [36]. The material properties, including Young’s modulus, were assigned by relating Hounsfield units (HU) to mechanical properties based on established empirical relations [37] (Fig. 2: Colored material mapped finite element meshes of an osteoporotic and a healthy specimen). This step ensured that subject-specific bone density and structure variations, resulting from different bone health conditions, were accurately represented in the FEA. The inferior surface of the vertebra was fixed in all directions, and an axial displacement of 0.5 mm was applied to the superior surface to simulate a quasi-compressive load and observe simulated fracture. After the simulation was carried out, the force–displacement diagram was derived, where the peak was defined as FL. The finite element simulation pipeline has previously been used and validated in multiple studies [25, 37–40].

### Statistics

The statistical analyses were performed with SPSS software (version 29; IBM SPSS Statistics for Windows, IBM Corp., Armonk, NY, USA). Tests were performed using a two-sided level of significance of 0.05. All values are given as mean ± standard deviation. Grubbs outlier test was performed to detect positioning inaccuracies by testing the ratio of attenuation signal acquired in horizontal and vertical positions; therefore, one vertebra was excluded. The Shapiro Wilk test showed significant differences from normal distribution for BMD and FEA-estimated FL. Correlations between attenuation, dark-field signals in horizontal and vertical positions, and FEA-estimated FL were tested using Spearman correlation. Additionally, the ratio of the signals acquired in vertical and horizontal positions was correlated with FEA-estimated FL. Differences in attenuation and dark-field signal of vertebrae were tested using a paired t-test. Differences in attenuation and dark-field signal of vertebrae with and without osteoporosis/osteopenia (BMD < 120 mg/cm^3^) were tested using the Wilcoxon Mann–Whitney U test. Differences in BMD and FEA-estimated FL of vertebrae with and without osteoporosis/osteopenia (BMD < 120 mg/cm^3^) were tested using an independent t-test.

The ability of FEA-estimated FL values to distinguish between vertebrae with and without osteoporosis/osteopenia was assessed using receiver-operating characteristics (ROC) curves, the respective area under the curve (AUC), and Youden’s Index to determine the optimal cutoff point.

## Results

### Specimens

We studied 58 vertebrae from spine specimens from 20 donors (9 men, 11 females) [20]. The average age of the donors was 73 years ± 13 (standard deviation), the average weight was 87 kg ± 29, and the average height was 165 cm ± 8 [20]. The non-osteopenic/osteoporotic group consisted of 11 patients (6 men) with an average age of 69 years ± 13, an average weight of 97 kg ± 29, an average height of 166 cm ± 7, and an average BMI of 35 kg/m^2^ ± 11 [20]. The osteoporotic/osteopenic group consisted of 9 individuals (6 women) with an average age of 78 years ± 11, an average weight of 74 kg ± 21, an average height of 163 cm ± 10, and an average BMI of 27 kg/m^2^ ± 5 [20]. There were no significant differences between the groups (*p* > 0.05) [20].

### BMD and FEA-estimated FL

**Fig. 3:**
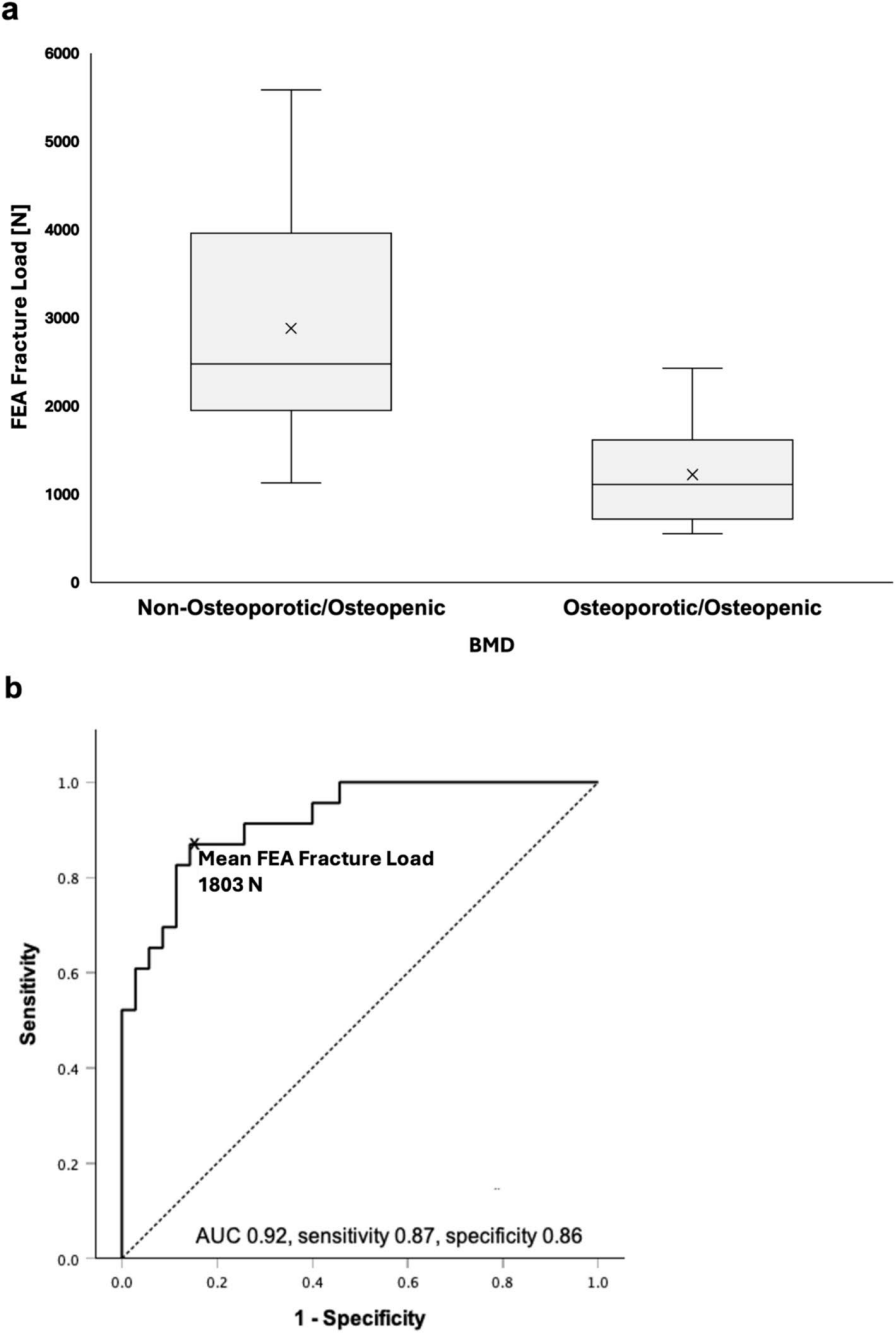
Quantitative analysis of the ability of CT-based finite element analysis (FEA) estimated fracture load to differentiate between osteoporotic/osteopenic vertebrae (BMD < 120 mg/cm^3^) and vertebrae with normal bone mineral density (BMD). **a** FEA-estimated fracture load differed significantly between the two groups (*p* < .001). **b** A receiver operating characteristic curve showed that the FEA-estimated fracture load was able to differentiate between osteoporotic/osteopenic vertebrae and those with normal BMD. The optimum cutoff value for FEA-estimated fracture load was 1803 N

The mean BMD across all vertebrae (*n* = 58) was 142 ± 59 mg/cm3; in the osteoporotic/osteopenic subgroup (*n* = 23) 75 ± 20 mg/cm3, in the group without osteoporosis/osteopenia (*n* = 35) 184 ± 28 mg/cm3 (*p* < 0.001) [20]. In the osteoporotic/osteopenic group, 11 vertebrae were osteoporotic (mean BMD < 80 mg/cm3) and 12 osteopenic (mean BMD < 120 mg/cm3) [20]. The mean FEA-estimated FL of all vertebrae was 2223 ± 1275 N. The mean FEA-estimated FL of osteoporotic/osteopenic vertebrae was significantly lower than that of vertebrae without osteoporosis/osteopenia (1222 ± 566 N vs. 2880 ± 1182 N, *p* < 0.001) (Fig. 3A). A positive correlation was found between BMD and FEA-estimated FL (r = 0.75, *p* < 0.001). The AUC for the FEA-estimated FL for differentiation of vertebrae with and without osteoporosis/osteopenia was 0.92 (Fig. 3B). The optimum cutoff value for FEA-estimated FL was 1803 N (sensitivity 0.87, specificity 0.86), the respective Youden index (J) was 0.73.

### Dark-field signal, attenuation signal and FEA-estimated FL

The mean dark-field signal across all vertebrae differed from 0.27 ± 0.06 in the vertical position to 0.32 ± 0.06 in the horizontal position (*p* < 0.001) [20]. The average mean attenuation signal was 0.27 ± 0.09 in vertical and horizontal position (*p* = 0.94) [20]. The average signal ratio between the signals acquired in vertical and horizontal position was 1.00 ± 0.07 for the attenuation signal and 0.84 ± 0.07 for the dark-field signal [20].

**Fig. 4:**
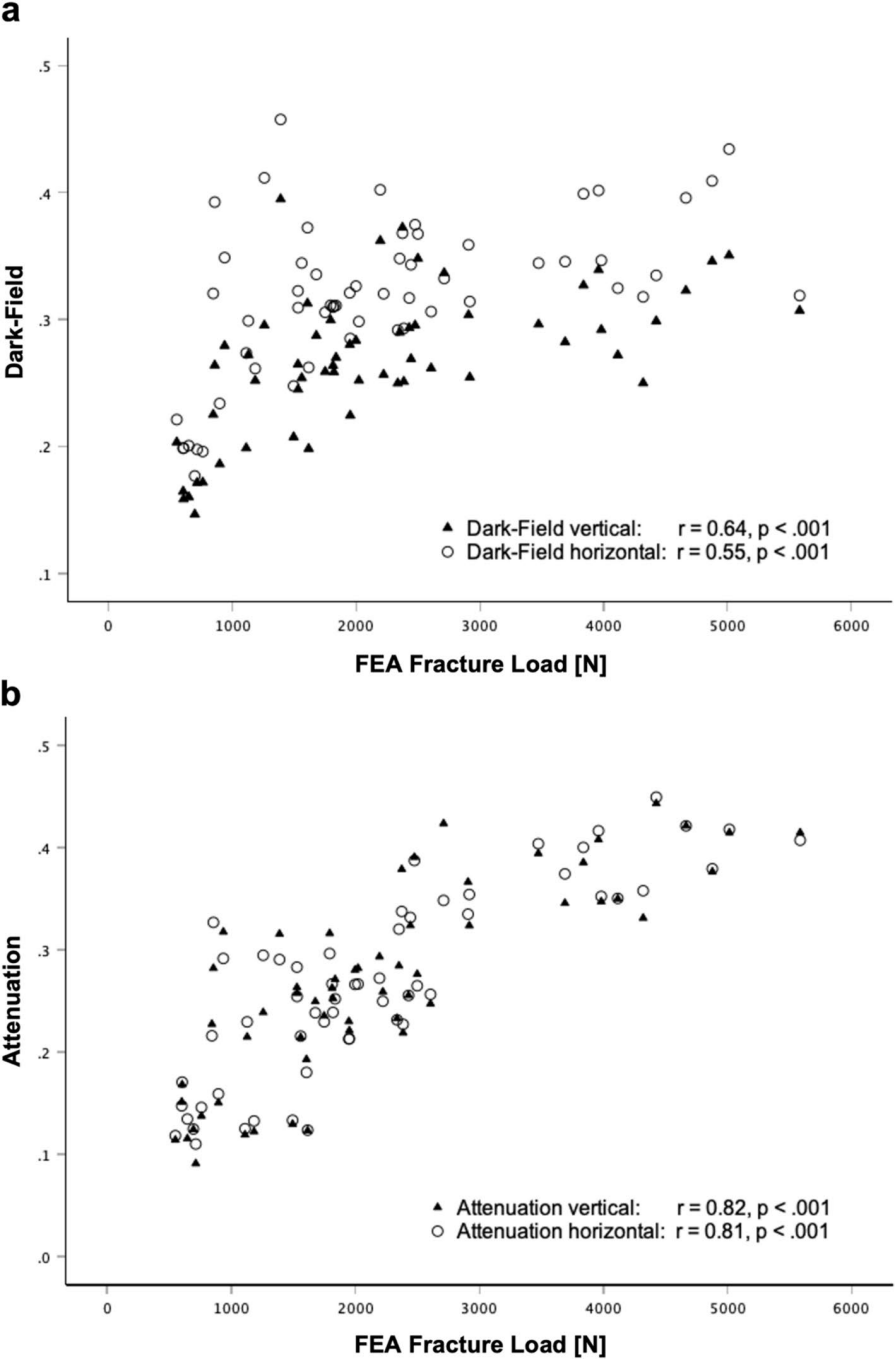
Statistical Analysis of the dark-field and attenuation signals of 58 vertebrae from 20 specimens. **a** Correlation between dark-field signal and FEA-estimated fracture load derived from CT. There was a moderate correlation between FEA-estimated fracture load and dark-field signal in vertical (r = 0.64, *p* <.001) and horizontal orientation (r = 0.55, *p* <.001). **b** Correlation between attenuation signal with FEA-estimated fracture load derived from CT. There was a strong correlation between FEA-estimated fracture load and attenuation signal in both vertical (r = 0.82, *p* <.001) and horizontal position (r = 0.81, *p* <.001)

The attenuation (ATT) and dark-field (DF) signals were positively correlated with the FEA-estimated FL, both in vertical (r_ATT/FL_ = 0.82, *p* < 0.001; r_DF/FL_ = 0.64, *p* < 0.001) and horizontal position (r_ATT/FL_ = 0.81, *p* < 0.001; r_DF/FL_ = 0.55, *p* < 0.001) (Fig. 4: Correlation between FEA-estimated fracture load and dark-field and attenuation signals). No correlation was found between the signal ratio of the attenuation signal, the signal ratio of the dark-field signal, and the FEA-estimated FL (r_ATT/FL_ = 0.12, *p* = 0.12; r_DF/FL_ = 0.17, *p* = 0.19).

## Discussion

In this prospective study, we examined the relationship between dark-field and attenuation signals derived from a clinical prototype for dark-field radiography and CT based FEA-estimated FL for 59 human cadaveric spine specimens with and without osteoporosis/osteopenia. The FEA-estimated FL was significantly lower in osteopenic/osteoporotic vertebrae compared to non osteopenic/osteoporotic vertebrae. The analysis revealed that the FEA-estimated FL correlated moderately with the dark-field signal acquired in horizontal and vertical orientations. There was a strong correlation between FEA-estimated FL and attenuation signal in horizontal and vertical orientations.

The dark-field prototype system is only sensitive to material interfaces in horizontal orientation due to the orientation of the gratings of the Talbot-Lau-Interferometer. Although the dark-field system is unable to resolve the trabecular structures of bone themselves, it can provide an estimation of the relative amount of trabeculae and bone strength. To evaluate the directionality of trabecular loss in osteoporosis, the vertebral specimens are scanned in horizontal and vertical positions. In previous studies, a higher dark-field signal in horizontal position compared to the signal in vertical position suggested a higher number of trabeculae in vertical orientation [18, 20, 41–43]. A positive correlation was shown between the ratio of dark-field signal in vertical and horizontal position and the BMD, indicating a lower ratio in osteopenic/osteoporotic vertebrae due to the reduced horizontally aligned trabeculae [20, 21]. For adaption to daily required loads, osteoporotic bone shows a dominant alignment in the vertical direction and a lack of trabeculae in the transverse direction [42, 44–46]. Moreover, microstructural bone parameters derived from micro-CT correlate with the dark-field signal [21]. For instance, the degree of anisometry negatively correlated with the dark-field signal only in the vertical position, not in the horizontal position, indicating that dark-field imaging can effectively detect characteristic changes in bone microarchitecture associated with osteoporosis [21].

As expected, the FEA-estimated FL was significantly lower in osteopenic/osteoporotic vertebrae compared to non osteopenic/osteoporotic vertebrae due to the decreased bone mass and compromised trabecular microstructure [40]. FEA-estimated FL classification achieved high sensitivity and specificity in differentiating vertebrae with osteoporosis/osteopenia from those with normal BMD. This finding confirms FEA-estimated FL as a surrogate for the mechanical strength of vertebrae. However, while FEA based FL analysis shows promise in assessing bone strength and fracture risk, it relies on volumetric CT imaging for accurate bone modelling. The added complexity, radiation exposure, accessibility, and the availability of alternative diagnostic methods may limit FEA’s widespread adoption for routine diagnosis of osteoporosis [40]. By using the dark-field signal as an indicator for the total number of structures present, it appears reasonable that a higher dark-field signal resembles a higher FEA-estimated FL. This is in line with results of Eggl et al., who reported that the combination of the dark-field signal in horizontal position and BMD predicted vertebral failure load derived from biomechanical testing significantly better than BMD alone, using an experimental dark-field set up [19]. In our study, no correlation was found between the ratio of the dark-field signal in vertical and horizontal position with the FEA-estimated FL. This may be due to the complex nature of bone strength, which is influenced by factors beyond just trabecular alignment and not fully captured by the dark-field signal ratio alone.

The attenuation signal showed an even stronger correlation with the FEA-estimated FL than the dark-field signal in both positions. This is unsurprising, given the previously demonstrated strong correlation between the attenuation signal and BMD, which in turn directly correlates with FEA-estimated FL in our study [20]. In-vivo, the attenuation signal of the spine would be superimposed with the attenuation of the surrounding tissue and, therefore, cannot be used to assess BMD. The current clinical standard for measuring bone strength, DXA, also uses attenuation information, yet, in a dual energy technique. However, DXA has been shown to be inadequate for BMD assessment primarily due to issues such as superimposing degenerative posterior elements of vertebrae [12].

The dark-field signal has the advantage of not being affected by interference from overlying soft tissue and of being selectively sensitive to material interfaces in horizontal orientation, the trabecula orientation affected most by osteoporosis. Dark-field imaging information on trabecular bone microstructure may, therefore, complement or augment BMD- or FEA-based approaches for predicting fracture risk, pending further in vivo validation. While not intended to replace the previously mentioned methods, dark-field imaging may ultimately serve as an accessible, dose-efficient technique to assess bone health, warranting further exploration. Compared to qCT, which typically involves effective doses on the order of 1–3 mSv for lumbar spine exams, our dark-field imaging prototyp operates at a dose closer to that of a single projection x-ray (~ 0.05–0.2 mSv, depending on acquisition parameters) [21, 47]. Moreover, although dark-field is inherently a 2D technique, scanning in multiple orientations can capture trabecular anisotropy by highlighting direction-dependent scattering signals. This partial directionality is both a strength (as it reveals trabecular alignment) and a limitation (since it does not provide full 3D data). Future enhancements may allow adaptation of dark-field imaging for other skeletal sites, particularly those where trabecular alignment is clinically relevant, such as the femoral neck. Dark-field radiography offers the advantage of retrofitting regular X-ray machines with the technology by adding grid lamellae. As our scanner is still a prototype, future research will be needed to define the specific modifications required for integrating this technology into existing X-ray systems.

This study has several limitations. We did not account for the potential impact of varying sample thicknesses on the measured signal. Due to the limited number of specimens in the osteoporosis/osteopenia group, we decided not to perform separate analyses for osteoporotic and osteopenic vertebrae, as this could lead to unstable and potentially misleading results. Future studies including larger and more diverse cohorts are needed to build on these preliminary findings and allow for more robust, subgroup-specific analyses. Lastly, the results from this ex-vivo study must be further investigated in in-vivo studies in the future.

In conclusion, our findings demonstrate that dark-field and attenuation signal from a prototype dark-field X-ray system correlate positively with CT based FEA-estimated FL, suggesting that dark-field imaging can provide structural information relevant to bone strength. While not intended to replace FEA, dark-field imaging may serve as a dose-efficient, accessible technique to complement existing methods for assessing osteoporosis-related bone fragility.

## Data Availability

The raw data supporting the conclusion of this article will be made available by the corresponding authors, without undue reservation, on reasonable request.

## Abbreviations

DXA: Dual-energy X-ray absorptiometry
qCT: Quantitative computed tomography
BMD: Bone mineral density
3D: Three-dimensional
FEA: Finite element analysis
FL: Fracture Load
HU: Hounsfield Unit
ROI: Region of interest
ROC: Receiver-operating characteristics
AUC: Area under the curve
